# Association of Intergenic and Intragenic *MGMT* Enhancer Methylation with *MGMT* Promoter Methylation, MGMT Protein Expression and Clinical and Demographic Parameters in Glioblastoma

**DOI:** 10.3390/ijms26073390

**Published:** 2025-04-04

**Authors:** Katharina Pühringer, Philipp Czarda, Sebastian Iluca, Katja Zappe, Serge Weis, Sabine Spiegl-Kreinecker, Margit Cichna-Markl

**Affiliations:** 1 Department of Analytical Chemistry, Faculty of Chemistry, University of Vienna, 1090 Vienna, Austria; katharina.puehringer@univie.ac.at (K.P.); philipp.czarda@outlook.com (P.C.); a11712252@unet.univie.ac.at (S.I.); katja.zappe@univie.ac.at (K.Z.); 2 Vienna Doctoral School in Chemistry (DoSChem), University of Vienna, 1090 Vienna, Austria; 3 Division of Neuropathology, Department of Pathology and Molecular Pathology, Kepler University Hospital GmbH, Johannes Kepler University, 4040 Linz, Austria; serge.weis@kepleruniklinikum.at; 4 Clinical Research Institute for Neurosciences, Johannes Kepler University, 4020 Linz, Austria; sabine.spiegl-kreinecker@kepleruniklinikum.at; 5 Department of Neurosurgery, Kepler University Hospital GmbH, Johannes Kepler University, 4040 Linz, Austria

**Keywords:** glioblastoma, MGMT, enhancer methylation, intergenic enhancer, intragenic enhancer, overall survival, prognostic biomarker, pyrosequencing

## Abstract

The methylation status of the *MGMT* gene promoter is recognized as a key predictive biomarker for glioblastoma patients, influencing treatment decisions and outcomes. Emerging evidence suggests that enhancer methylation may also play a role in gene regulation and is associated with various clinical parameters, genetic variants, and demographic factors. This study aimed to assess DNA methylation levels in intergenic and intragenic *MGMT* enhancers to investigate their relationship with *MGMT* promoter methylation, MGMT protein expression, and clinical and demographic characteristics in glioblastoma. We developed 18 pyrosequencing assays to analyze 54 CpGs, including 34 in intergenic and 20 in intragenic enhancers. The assays were applied to tumor cells derived from 38 glioma patients. Intragenic enhancer CpGs showed significantly higher methylation than intergenic enhancer CpGs. Intragenic enhancer methylation showed a strong negative correlation with *MGMT* promoter methylation. For several CpGs in intragenic enhancers, an inverse L-shaped relationship between methylation levels and MGMT expression was observed. We identified distinct associations between enhancer methylation and clinical and demographic parameters. Intergenic enhancer methylation was primarily linked to the *TERT* SNP rs2853669 genotype, Ki-67 expression, age, and sex, whereas intragenic enhancer methylation was associated with *MGMT* promoter methylation, MGMT expression, overall survival, and progression-free survival. Further studies with larger patient cohorts are needed to validate the clinical relevance of intergenic and intragenic *MGMT* enhancer methylation in glioblastoma.

## 1. Introduction

The DNA repair protein *O*6-methylguanine DNA methyltransferase (MGMT) is essential for maintaining genome integrity by removing alkylating lesions at the O6 position of guanine [1]. MGMT is expressed in various normal tissues, however, MGMT expression has also been reported for several tumor types [2,3]. High MGMT expression in tumor tissues compromises the efficacy of chemotherapy with DNA alkylating agents, such as temozolomide (TMZ) [4,5]. TMZ is a key component of the standard therapy for patients diagnosed with glioblastoma [6]. Glioblastoma, defined as isocitrate dehydrogenase (IDH)-wild-type diffuse astrocytoma exhibiting distinct genetic and epigenetic alterations [7], is highly aggressive and accounts for approximately 45–50% of all primary malignant brain tumors in adults. Genetic abnormalities include mutations in the telomerase reverse transcriptase (*TERT*) promoter, gene amplification of the epidermal growth factor receptor (*EGFR*), and co-occurrence of chromosome 7 gain and chromosome 10 loss [8]. Dysregulation of epigenetic marks, such as DNA methylation and histone modifications, contributes to glioblastoma development and inter-patient heterogeneity, highlighting their potential as targets for personalized epigenetic therapies [9,10].

The response of glioblastoma patients to TMZ is critically influenced by the DNA methylation status of the *MGMT* promoter [11]. Hypermethylation of the *MGMT* promoter, resulting in transcriptional silencing, has been associated with improved survival outcomes from TMZ therapy [12]. Consequently, the *MGMT* promoter methylation status serves as a predictive biomarker for TMZ response, in particular in elderly patients with newly diagnosed glioblastoma [13]. Among the 98 CpG dinucleotides (CpGs) in the CpG island, the methylation status of CpGs 72–83 has been identified as particularly meaningful. However, discrepancies exist wherein some tumors express MGMT despite promoter methylation, while others lack MGMT expression despite promoter unmethylation [14]. This suggests that additional regulatory mechanisms influence MGMT protein expression. Recent findings indicate that methylation of CpGs in *MGMT* enhancers is associated with MGMT expression in glioblastoma [15].

Enhancers are regulatory elements that are crucial for the spatial and temporal control of gene expression [16]. They play a central role in normal development and in responding to complex environmental signals [17]. Unlike promoters, enhancers can activate transcription of their target genes over great distances, ranging from several to hundreds or even thousands of kilobases [18]. On average, enhancers are 200–500 base pairs (bp) in length, contain recognition sites for multiple transcription factors [19,20], and are characterized by a low density of CpGs [21]. Aberrations in enhancer methylation have been linked to various diseases, including cancer [22,23,24,25]. Evidence suggests that enhancers are the most differentially methylated regions during cancer progression from normal to primary tumors and subsequently to metastases [17]. Thus, enhancer methylation may serve as a potential prognostic and/or predictive biomarker for various types of cancer [15,26,27]. In cancers, gene expression may be better predicted by enhancer methylation than by promoter methylation, likely because enhancers undergo earlier and more dynamic epigenetic changes, influencing gene regulation over long genomic distances [28].

In a previous study, we analyzed the methylation status of CpGs in three intergenic enhancers and one intragenic enhancer using in-house developed pyrosequencing (PSQ) assays [15]. Our findings suggested significant differences in absolute DNA methylation levels between intergenic and intragenic enhancers and their associations with MGMT expression [15], clinical parameters, genetic variants, and demographic characteristics in glioblastoma [27].

In this follow-up study, we aimed to further investigate the role of DNA methylation in *MGMT* enhancers. We therefore developed PSQ assays targeting previously unexamined *MGMT* enhancers [15] and additional CpGs within previously analyzed enhancers that were not covered by our previous assays [15]. To obtain a more comprehensive view, we integrated methylation data for a sample set consisting of tumor cells derived from 38 glioma patients from this study and our previous study [15] and investigated their associations with MGMT expression, as well as with multiple clinical and demographic parameters, including proliferation index Ki-67, overall survival (OS), long-term survival, progression-free survival (PFS), postoperative Karnofsky Performance Score (KPS), *TERT* promoter mutations C228T and C250T, *TERT* SNP rs2853669, age, and sex of the patients.

## 2. Results

In our previous study [15], we reported CpG methylation levels for three intergenic enhancers (hs737 [29]; the enhancer identified by Chen et al. [30]; and hs699 [29]) and one intragenic enhancer (hs696 [29]). In this study, we analyzed the DNA methylation status of 35 CpGs in previously unexamined enhancers, including two intergenic enhancers (hs542, hs562 [29]) and three intragenic enhancers (hs656, hs331, hs589 [29]), as well as additional CpGs in previously examined enhancers. For clarity, we established an internal enhancer nomenclature using capital letters and ordering the enhancers based on their genomic position. Enhancers A (hs542 [29]), B (hs737 [29]), C (the enhancer identified by Chen et al. [30], D (hs699 [29]), and E (hs562 [29]) are intergenic enhancers, located between the *Ki-67* and the *MGMT* genes. In contrast, enhancers F (hs656 [29]), G (hs696 [29], H (hs331 [29]), and I (hs589 [29]) are intragenic enhancers, with enhancers F, G, and H located in intron 2 and enhancer I being in intron 3 of the *MGMT* gene.

**Figure 1:**
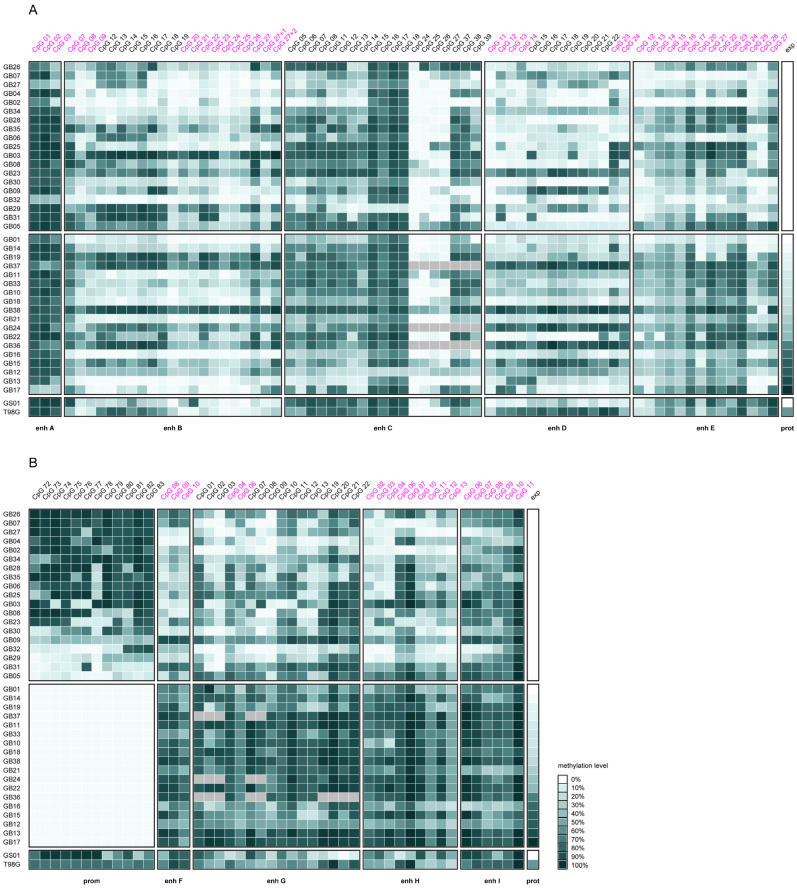
Heatmaps of CpG methylation levels in (**A**) intergenic and (**B**) intragenic *MGMT* enhancers and the *MGMT* promoter (CpGs 72–83) in 37 glioblastoma (GB01–19 and GB21–38), one gliosarcoma (GS01), and the commercial glioblastoma cell line T98G. Enhancers are sorted according to their position in the genome. The two panels display *MGMT* promoter methylated (top) and unmethylated (bottom) samples. Samples lacking *MGMT* promoter methylation are sorted by MGMT expression levels, determined by Western blot analysis and given relative to the MGMT overexpressing glioblastoma cell line GL80 [14]. Methylation levels are the mean of two individual PSQ runs. For DNA methylation levels as well as MGMT protein expression, the color scale is in the range of 0–100% (white to dark green), where the highest relative MGMT expression level of 1.32 (GB17) was set to 100%. CpGs analyzed in the current study are highlighted in pink. Missing values are colored grey.

Heatmaps indicating methylation levels of individual CpGs in enhancers A–I and the *MGMT* promoter and MGMT protein expression levels for patient-derived tumor cells (PDCs) from 37 glioblastoma patients and one gliosarcoma patient and the commercially available glioblastoma cell line T98G are shown in Figure 1. CpGs targeted in this study are highlighted in pink.

### 2.1. Overall Intergenic and Intragenic Enhancer Methylation

**Figure 2:**
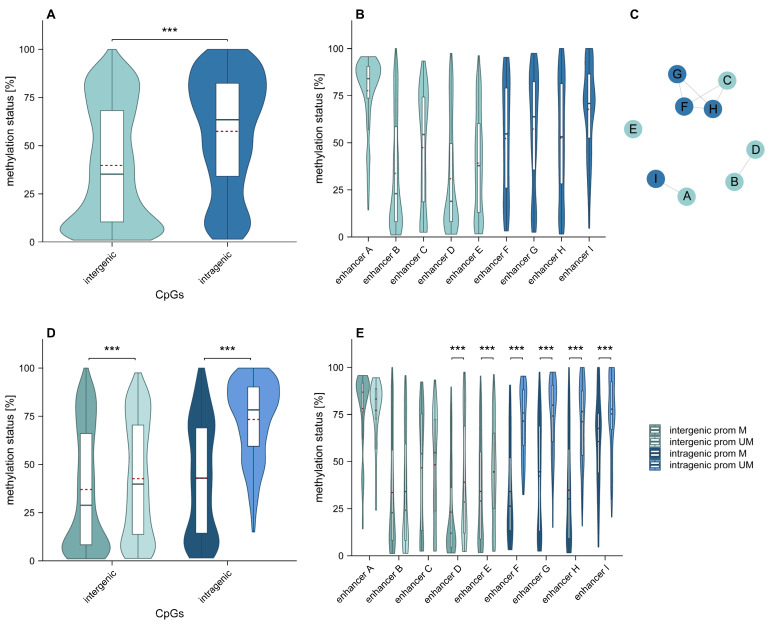
(**A**–**E**) Distribution of methylation levels in intergenic (turquoise) and intragenic (blue) enhancers at CpG sites analyzed in this study and in our previous study [15]. Included samples: GB01–19 and GB21–38. Red dashed line indicates the arithmetic mean. *** *p* ≤ 0.001. (**A**) Methylation levels for intergenic and intragenic CpGs. (**B**) Methylation levels for individual enhancers. (**C**) Network graph. (**D**) Methylation levels for all CpGs included, stratified by *MGMT* promoter methylation status (promoter unmethylated/methylated shown in light/dark hues, respectively). (**E**) Methylation levels for individual enhancers stratified by the *MGMT* promoter methylation status. prom: promoter, M: methylated, UM: unmethylated.

Overall, intragenic CpGs showed significantly higher (*p* < 0.001) methylation than intergenic CpGs (Figure 2A). In addition, we identified several significant differences in methylation levels among individual enhancers. To illustrate these differences clearly, we created a network graph highlighting similarities in methylation levels. Enhancers B and D; C, F, G, and H; and A and I showed similar methylation patterns (Figure 2B,C). Our results indicate that *MGMT* promoter unmethylated samples showed significantly higher methylation (*p* < 0.001) of both intergenic and intragenic enhancers compared to promoter methylated samples (Figure 2D). Significant differences in methylation levels between *MGMT* promoter methylated and unmethylated samples were found for intergenic enhancers D and E, and intragenic enhancers F, G, H, and I (*p* < 0.001) (Figure 2E).

### 2.2. Association of Intergenic and Intragenic Enhancer Methylation with the Relative Position of CpGs

**Figure 3:**
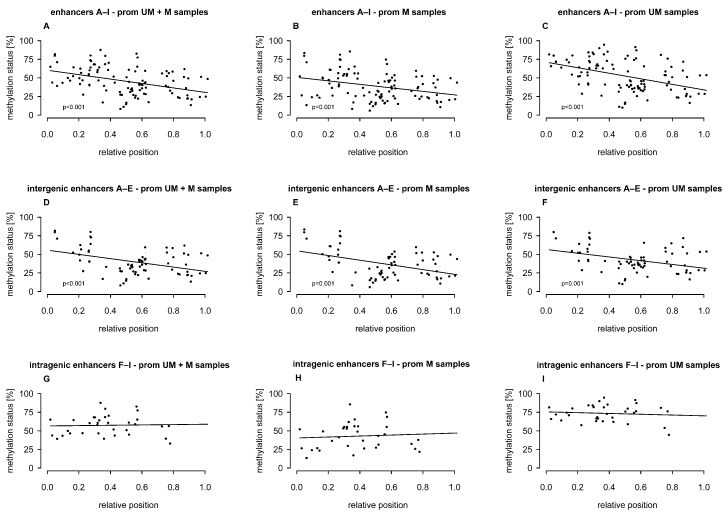
Association of intergenic and intragenic enhancer methylation levels with the relative position of CpGs in the enhancers A–I, including methylation data from this study and from our previous study [15]. Included samples: GB01–19 and GB21–38. (**A**–**C**) Enhancers A–I, (**D**–**F**) intergenic enhancers A–E, (**G**–**I**) intragenic enhancers F–I. prom: promoter, M: methylated, UM: unmethylated.

In general, CpG density in the enhancers was relatively low, ranging from 1.6% (enhancer A) to 4.8% (enhancer B). We investigated whether methylation levels were associated with the relative position of the CpGs in the enhancers A–I, including methylation levels at CpGs analyzed in this study and in our previous study [15]. When examining enhancers individually, no meaningful associations were found. However, when taking into account CpGs from all enhancers or from intergenic enhancers only, we found that CpGs closer to the 5′ end showed significantly higher methylation than those closer to the 3′ end. This trend was consistent regardless of whether samples were stratified by *MGMT* promoter methylation status. In contrast, the methylation status of CpGs in intragenic enhancers did not show an association with their relative position in the enhancer (Figure 3A–I).

### 2.3. Association Between MGMT Promoter and Enhancer Methylation

Correlation analysis was performed to evaluate associations between CpGs in the same or different enhancers, as well as between CpGs in enhancers and those in the *MGMT* promoter. To obtain a more comprehensive view, CpG methylation levels from this study and from our previous study [15] were included. The analysis was performed on samples both with and without stratification by the *MGMT* promoter methylation status.

**Figure 4:**
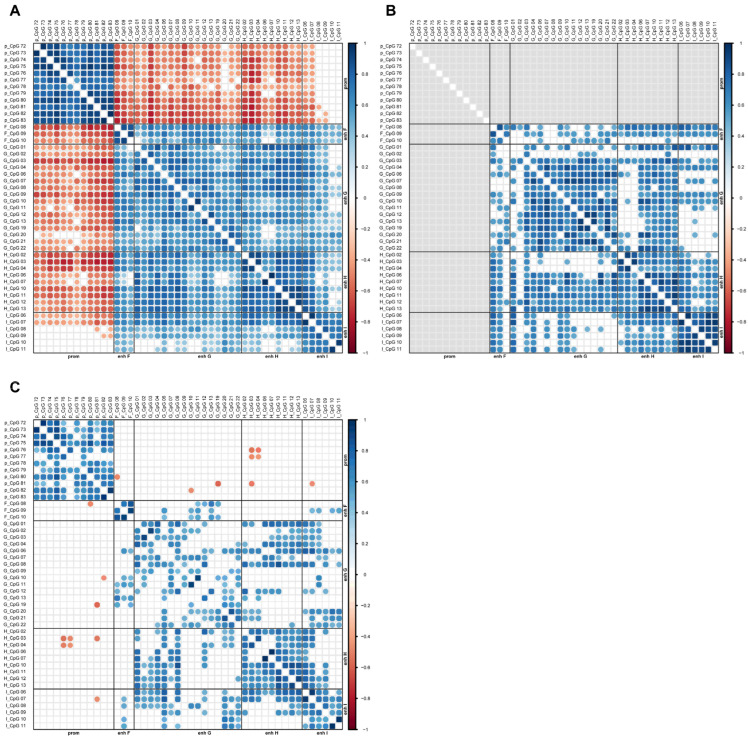
Correlation plots displaying significant Pearson’s correlation coefficients between methylation levels of individual CpGs in intragenic *MGMT* enhancers and the *MGMT* promoter. CpG methylation levels from this study and from our previous study [15] were included. The color code ranges from dark red (−1.0) to dark blue (1.0). Grey tiles indicate non-analyzable data. Plots are shown for (**A**) all, (**B**) *MGMT* promoter unmethylated, and (**C**) *MGMT* promoter methylated glioblastoma (GB01–19 and GB21–38). Correlation analysis was carried out using pairwise complete comparisons.

Correlation plots between CpGs in intragenic enhancers are shown in Figure 4. 

For intragenic enhancers F, G, H, and I, the most and strongest positive correlations between CpGs were observed when samples were not stratified by the *MGMT* promoter methylation status (Figure 4A). Within enhancers F, H, and I, almost all CpGs correlated strongly with each other. This holds true for samples with unmethylated promoters as well as for samples without stratification. When considering all glioblastoma samples, strong negative correlations were found between *MGMT* promoter CpGs 72–83 and all CpGs in enhancer F, as well as nearly all investigated CpGs in enhancers G and H. In addition, methylation of all twelve *MGMT* promoter CpGs correlated with methylation of CpGs 06 and 07 in enhancer I, although this was not or not to the same extent observed for CpGs 08–11. Correlations between CpGs from different intragenic enhancers were found across all enhancers, with the highest number of correlations observed without sample stratification (Figure 4A). 

Correlation plots for intergenic CpGs are shown in Supplementary Figure S1. Within intergenic enhancers B and D, all CpGs correlated with each other, respectively, when taking into account all, or promoter unmethylated samples only. In contrast to intragenic enhancers, hardly any negative correlations between *MGMT* promoter CpGs and intergenic CpGs were seen.

### 2.4. Association of MGMT Promoter and Enhancer Methylation with MGMT Protein Expression

Since sample GB01 lacked MGMT expression despite an unmethylated *MGMT* promoter, we concluded that DNA methylation did not play a role in MGMT expression in this sample [15]. Consequently, GB01 was excluded from the statistical analysis on enhancer methylation and MGMT expression. Our analysis was thus confined to GB02–19 and GB21–38. Methylation data from this study and from our previous study [15] were included to obtain a more comprehensive view on the association of *MGMT* promoter and enhancer methylation with MGMT protein expression.

**Figure 5:**
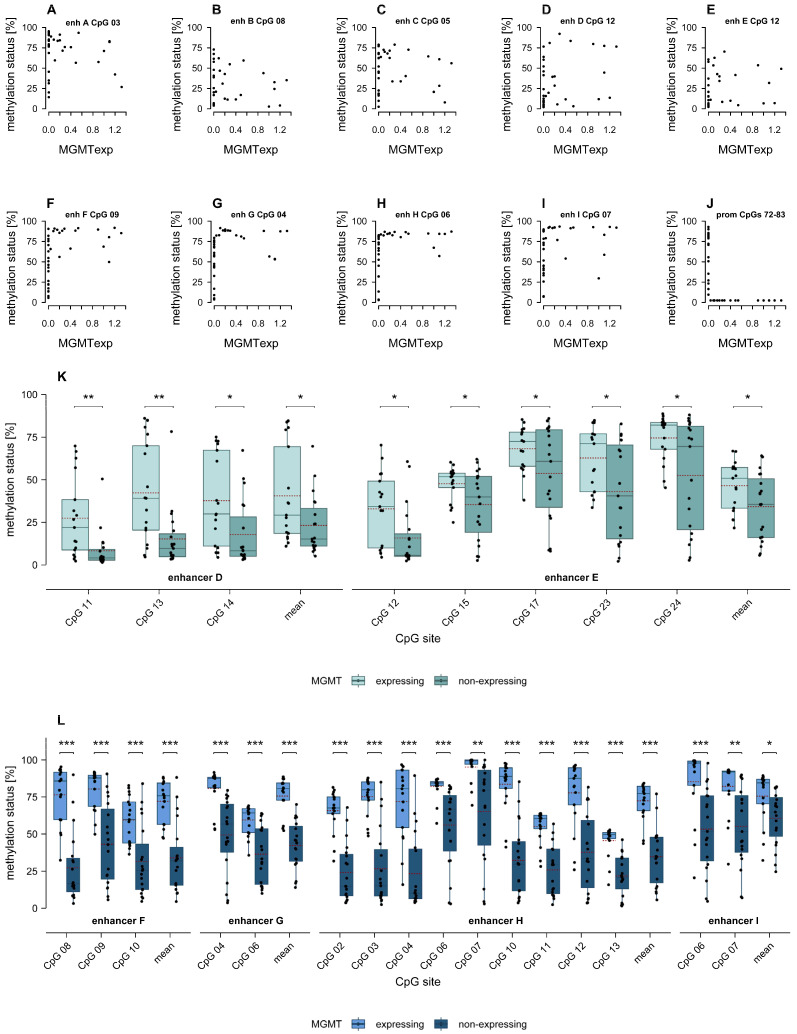
(**A**–**J**) Association between enhancer/promoter methylation and MGMT protein expression. (**A**–**E**) Intergenic enhancers, (**F**–**I**) intragenic enhancers, (**J**) promoter. (**C**) Methylation data for enhancer C CpGs as well as for the promoter was acquired in our previous study [15]. (**K**,**L**) Boxplots showing significant differences in methylation levels in (**K**) intergenic and (**L**) intragenic *MGMT* enhancers between MGMT expressing (light) and non-expressing (dark) glioblastoma samples (GB02–19 and GB21–38). The red dashed line represents the mean methylation level. * *p* ≤ 0.05, ** *p* ≤ 0.01, *** *p* ≤ 0.001.

When investigating potential associations between enhancer methylation and MGMT expression, significant linear correlations were neither found for intergenic nor for intragenic enhancers (Figure 5A–I). 

However, we could see a trend of an inverse L-shaped relationship between enhancer methylation and MGMT protein expression for some CpGs located in intragenic enhancers, namely CpG 09 in enhancer F, CpGs 01, 04, 07, 10, 11, 20, and 22 in enhancer G, CpGs 02, 03, 06, 07, 10, 11, and 13 in enhancer H, and CpGs 06, 07, 10, and 11 in enhancer I. Exemplary plots are shown in Figure 5F–I. Specifically, MGMT was not expressed at low to intermediate methylation levels but was expressed at high methylation levels, with a few exceptions. This contrasts with the association between *MGMT* promoter methylation and expression (Figure 5J), where low methylation generally is sufficient to silence *MGMT*, and MGMT is typically expressed only when the promoter is unmethylated.

Boxplots in Figure 5K,L illustrate the distribution of methylation levels at individual CpGs in intergenic and intragenic enhancers, respectively. Samples are grouped according to their MGMT protein expression, which also implies the *MGMT* promoter methylation status. For CpGs located in enhancers A and B, there was no significant difference in methylation levels between MGMT expressing and non-expressing glioblastoma samples.

In enhancer D, MGMT expressing samples showed significantly higher methylation levels at three CpGs (*p* ≤ 0.017) as well as at mean methylation across CpGs 11–24 (*p* = 0.035). At five CpGs in enhancer E, methylation levels were also higher in MGMT expressing glioblastoma samples compared to non-expressing ones (*p* ≤ 0.05). Overall, mean methylation across CpGs 12–17 and 20–27 in enhancer E was also higher in MGMT expressing samples (*p* = 0.038).

In intragenic enhancers, both methylation levels at individual CpGs as well as the mean methylation levels across CpGs within the same enhancer were higher in MGMT expressing samples compared to non-expressing glioblastoma samples. This difference was significant for all individually analyzed CpGs in the present study as well as for the mean methylation across CpGs of the respective enhancers F, G, and H. Similarly, methylation levels of the first two analyzed CpGs (CpG 06 and CpG 07) as well as the mean methylation of CpGs 06–09 of enhancer I displayed significantly higher methylation levels in MGMT expressing samples compared to non-expressing ones (*p* ≤ 0.018). In contrast, methylation levels at CpGs 08–11 did not significantly differ between the two groups.

### 2.5. Association of MGMT Enhancer Methylation with Clinical and Demographic Parameters

We observed significant differences in methylation levels between immortalized and primo-cell cultures at several CpGs across all enhancers, with and without sample stratification, except intergenic enhancer A (see Supplementary Figure S2). In all cases, methylation levels were significantly higher in primo-cell culture samples than in immortalized ones. Therefore, primo-cell culture samples (GB36–38) were excluded from statistical analysis of clinical and demographic parameters. For the calculation of mean methylation levels across CpGs within enhancers B, D, and G, data from our previous study was included [27].

### 2.6. Association of MGMT Enhancer Methylation with Overall Survival, Progression-Free Survival and Long-Term Survival

**Figure 6:**
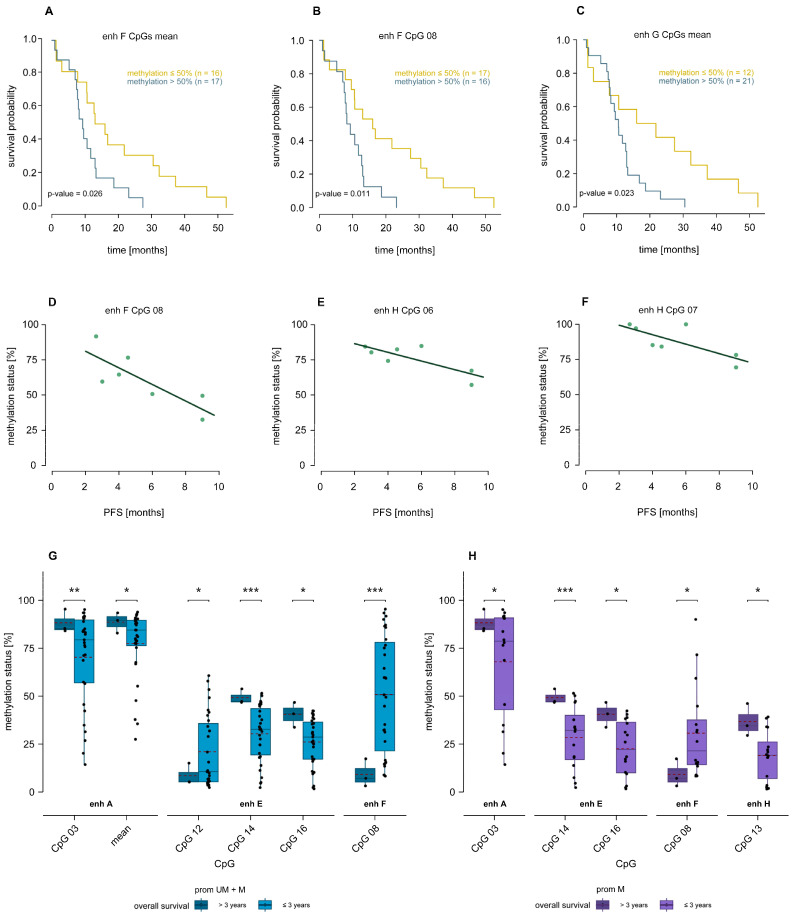
(**A**–**C**) Kaplan–Meier survival analysis. Glioblastoma samples were stratified by methylation status of (**A**) enhancer F mean (CpGs 08–10), (**B**) enhancer F CpG 08 and (**C**) enhancer G mean (CpGs 01–04, 06–13, and 19–22). GB27 was excluded from analysis due to lacking OS data. (Yellow: methylation below or equal cut-off level, blue: above cut-off level.) (**D**–**F**) Significant correlations between enhancer methylation and PFS, taking into account *MGMT* promoter unmethylated samples only. (**D**) CpG 08 in enhancer F, (**E**) CpG 06 and (**F**) CpG 07 in enhancer H. (**G**,**H**) Significantly different *MGMT* enhancer methylation levels between long-term survivors and short-term survivors including (**G**) all and (**H**) *MGMT* promoter methylated glioblastoma samples only. The red dashed line represents the mean methylation level. Significance marks: * *p* ≤ 0.05, ** *p* ≤ 0.01, *** *p* ≤ 0.001.

Among the intragenic enhancers, methylation levels of enhancer H and I were not predictive for overall survival in Kaplan–Meier survival analyses using cut-off levels ranging from 5–90%. However, by using a 50% cut-off level, mean methylation of the CpGs targeted in enhancer F (CpGs 08–10) or methylation of individual CpG 08 could predict overall survival (*p* = 0.026 and 0.011, respectively). In addition, mean methylation of enhancer G (CpGs 01–04, 06–13, and 19–22) was predictive for overall survival (*p* = 0.023). In all cases, patients with lower methylation had significantly longer overall survival compared to those with higher methylation (Figure 6A–C). 

Significant differences were also obtained by applying cut-off levels from 20–60% (*p* ≤ 0.048); however, in these cases, the two group sizes (high/low methylation) were relatively different. Methylation of intergenic enhancers showed no significant association with overall survival, regardless of stratification by the *MGMT* promoter methylation status.

We found significant negative correlations between intragenic enhancer methylation and progression-free survival. Specifically, we found a significant negative correlation for CpG 08 in intragenic enhancer F (*p* = 0.027, r = −0.810), and CpGs 06 (*p* = 0.034, r = −0.791) and 07 (*p* = 0.048, r = −0.759) in enhancer H, when only *MGMT* promoter unmethylated samples were considered (Figure 6D–F). Higher methylation levels at these CpGs were associated with shorter progression-free survival compared to lower methylation levels. In contrast, we did not find any association between progression-free survival and methylation of intergenic enhancers.

Three glioblastoma samples (GB03, GB04, and GB23) originated from long-term survivors, defined as patients who were still alive three years after surgery. We found differences in methylation levels at CpGs in intergenic enhancers A and E and intragenic enhancers F and H between long-term and short-term survivors (Figure 6G,H). Significantly higher methylation levels in long-term survivors compared to short-term survivors were found for mean methylation of CpGs 01–03 enhancer A when all samples were taken into account (*p* = 0.035). Conversely, significantly higher methylation levels were detected in short-term survivors compared to long-term survivors at CpG 12 in enhancer E (*p* = 0.029) and CpG 08 in enhancer F (*p* < 0.001) when all samples were included and at CpG 08 in enhancer F, when only promoter methylated samples were considered (*p* = 0.013). For CpG 03 in enhancer A and CpGs 14 and 16 in enhancer E, methylation levels were significantly higher in long-term survivors, regardless of whether all samples or only *MGMT* promoter methylated samples were included in the analysis (*p* ≤ 0.035). For CpG 13 in enhancer H, we found significantly higher methylation levels in long-term survivors, when only *MGMT* promoter methylated samples were included in the analysis (*p* = 0.039).

No meaningful correlation was identified between enhancer methylation and KPS, whether for intergenic or intragenic enhancers, and regardless of whether samples were stratified by the *MGMT* promoter methylation status.

### 2.7. Association of MGMT Enhancer Methylation with TERT SNP rs2853669 and TERT Promoter Mutations C228T and C250T

**Figure 7:**
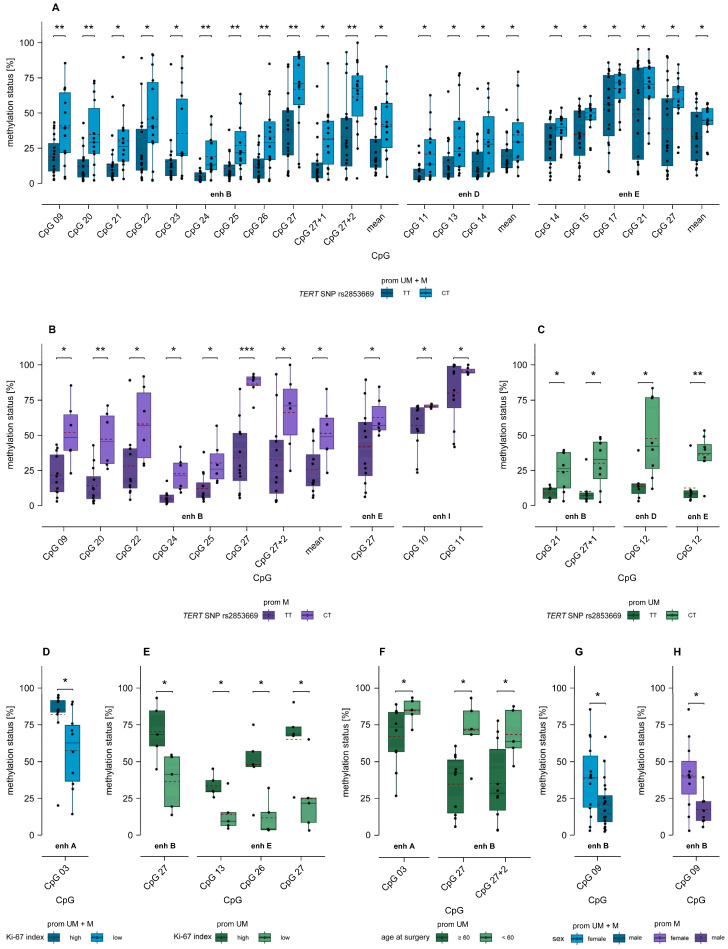
(**A**–**C**) Significantly different *MGMT* enhancer methylation levels between patients with *TERT* SNP rs2853669 wildtype (TT) and mutated (CT) genotype in (**A**) all, (**B**) promoter methylated, and (**C**) promoter unmethylated glioblastoma samples. For the calculation of mean methylation within the enhancers B and D, data from our previous study [27] were included. (**D**,**E**) Boxplots showing significant differences in methylation levels between glioblastoma samples with high and low Ki-67 index in (**D**) all and (**E**) promoter unmethylated samples. (**F**–**H**) Association between *MGMT* enhancer methylation and age and sex. (**F**) Significantly different *MGMT* enhancer methylation levels between patients < 60 years and patients ≥ 60 years at surgery in promoter unmethylated glioblastoma samples. Significantly different *MGMT* enhancer methylation levels between male and female patients in (**G**) all and (**H**) promoter methylated glioblastoma samples. The red dashed line represents the mean methylation level. Significance marks: * *p* ≤ 0.05, ** *p* ≤ 0.01, *** *p* ≤ 0.001.

Next, we examined differences in methylation levels between samples with either the wildtype (homozygous TT) or the mutated (heterozygous CT) genotype of *TERT* SNP rs2853669. Significantly higher methylation levels were observed at CpGs located in intergenic enhancers B, D, and E in samples with the mutated genotype (CT) compared to those with the wildtype genotype (TT), without sample stratification. More precisely, this held true for nine CpGs in enhancer B as well as the two additional CpGs located downstream of enhancer B, and mean methylation across all analyzed CpGs in enhancer B (*p* ≤ 0.043), three individual as well as mean methylation of CpGs in enhancer D (*p* ≤ 0.044), and five CpGs as well as mean methylation of analyzed CpGs in enhancer E (*p* ≤ 0.048) (Figure 7A).

When only promoter methylated glioblastoma samples were included, significant differences were found for a smaller number of CpGs: for six CpGs within enhancer B, one CpG downstream of enhancer B (CpG 27 + 2) as well as mean methylation across CpGs in enhancer B (*p* ≤ 0.048), one CpG (CpG 27) in enhancer E (*p* = 0.048), and two CpGs (CpG 10, 11) in enhancer I (*p* ≤ 0.035) (Figure 7B).

In promoter unmethylated samples with *TERT* SNP mutation, significantly higher methylation levels for the mutated genotype were observed at one CpG within (CpG 21) and one CpG downstream of enhancer B (CpG 27 + 1) (*p* ≤ 0.020), one CpG (CpG 12) in enhancer D (*p* = 0.013), and one CpG (CpG 12) in enhancer E (*p* = 0.006) (Figure 7C).

We identified significant differences in the methylation of CpG 14 in intergenic enhancer E based on *TERT* promoter genotypes. Specifically, samples with the C228T mutation exhibited significantly higher methylation levels compared to wildtype samples (*p* = 0.038), when all samples were included in the statistical analysis (Supplementary Figure S3).

### 2.8. Association of MGMT Enhancer Methylation with Ki-67 Index

We assessed whether *MGMT* enhancer methylation is associated with the proliferation index Ki-67. When we divided the glioblastoma samples into two groups based on Ki-67 index (≤50% (low) and >50% (high)), significantly higher methylation levels were observed at CpG 03 in intergenic enhancer A (*p* = 0.023) in samples with high Ki-67 index compared to those with low Ki-67 index (Figure 7D). Among glioblastoma samples with unmethylated *MGMT* promoter, significantly higher methylation levels were found in samples with high Ki-67 index at CpG 27 in enhancer B (*p* = 0.023), and CpG 13, 26, and 27 (*p* ≤ 0.029) in enhancer E (Figure 7E).

### 2.9. Association of MGMT Enhancer Methylation with Age and Sex

To investigate whether enhancer methylation is associated with age, patients were divided into two subgroups: those under 60 years and those aged 60 or older at the time of surgery. Significant differences in enhancer methylation between these two age groups were found exclusively for intergenic enhancers and only in *MGMT* promoter unmethylated glioblastoma samples. Specifically, one CpG in enhancer A (CpG 03; *p* = 0.037), one CpG in enhancer B (CpG 27), as well as one located downstream of enhancer B (CpG27 + 2; *p* ≤ 0.014) were significantly higher methylated in patients < 60 years compared to those ≥ 60 years (Figure 7F). At one single CpG (CpG 09 in intergenic enhancer B), the methylation status was significantly higher in female patients compared to male patients. This difference was observed both when analyzing all samples (*p* = 0.030) and when focusing on the subgroup of promoter methylated samples (*p* = 0.013) (Figure 7G,H).

## 3. Discussion

Evidence is accumulating that, in addition to methylation of gene promoters, methylation of enhancers plays a significant role in gene regulation [17,28]. Enhancers are cis-regulatory elements that ensure precise spatiotemporal control of gene expression [31]. Compared to promoters, enhancer-mediated regulation of gene expression is more complex. Multiple enhancers can regulate a single gene, while a single enhancer may control the expression of multiple genes. Although many enhancers are located in close proximity to the promoter they activate, some can be found at considerable distances (50–100 kb) from the transcription start site, whether upstream, downstream, or even within the target gene [32].

In this study, we aimed to explore the role of intergenic and intragenic enhancer methylation in MGMT expression as well as its association with clinical parameters, including overall survival, genetic variants, and demographic factors in glioblastoma. We mainly focused on intergenic and intragenic enhancers listed in the VISTA Enhancer Browser [29] because they are experimentally validated and known to be active in various tissues. In addition, we targeted an intergenic enhancer identified by Chen et al. [30], which has been associated with *MGMT* regulation.

We used a sample set comprising immortalized and primo-cell cultures derived from 38 gliomas as the model for our research.

Altogether, in this study and our previous work [15], we targeted a total of 105 CpGs in five intergenic (enhancers A, B, C, D, E) and four intragenic *MGMT* enhancers (enhancers F, G, H, I), utilizing in-house developed PSQ assays. Notably, only six of the CpGs targeted in this or our previous study [15] are covered by commercial microarrays. CpG 09 in enhancer F, CpGs 12 and 20 in enhancer G, and CpGs 14, 15 and 21 in enhancer D are targeted by Methylation EPIC v1.0 BeadChip (850k) and Infinium MethylationEPIC v2.0 BeadChip (935k).

In general, the CpG density of the enhancers was relatively low, ranging from 1.6% (enhancer A) to 4.8% (enhancer B). Interestingly, we found a relationship between methylation levels and the relative position of the CpGs within the enhancers, but this was observed exclusively for intergenic enhancers. In intergenic enhancers, CpGs closer to the 5′ end were significantly more methylated compared to those closer to the 3′ end, regardless of the *MGMT* promoter methylation status. This is contrary to what has been reported for CpGs in the first intron of genes, where unmethylated CpGs tend to be located closer to the 5′ end of the intron, while methylated CpGs are found further downstream [33]. However, to the best of our knowledge, these regions in intron 1 have not been identified as enhancers.

Overall, intragenic enhancer CpGs showed significantly higher methylation (*p* < 0.001) compared to intergenic enhancer CpGs. In addition, *MGMT* promoter unmethylated samples showed significantly higher methylation (*p* < 0.001) in intergenic enhancers D and E, as well as intragenic enhancers F, G, H, and I, compared to promoter methylated samples.

**Figure 8:**
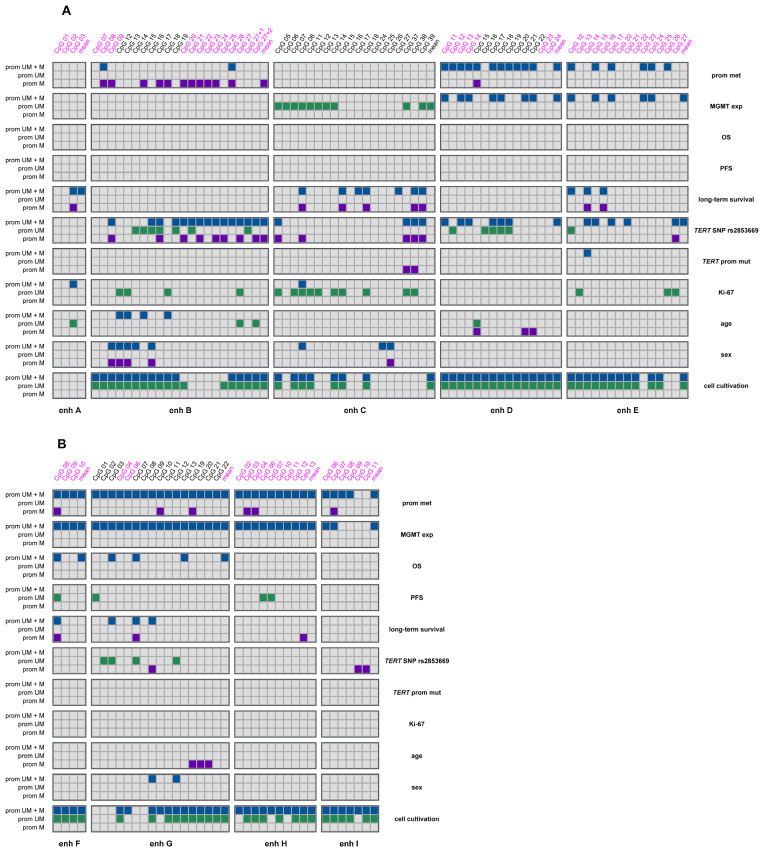
Summary of associations found for *MGMT* enhancer methylation in (**A**) intergenic enhancers, and (**B**) intragenic enhancers acquired in the current (marked in pink) and previous studies [15,27]. Significant associations are highlighted for prom M–promoter methylated (purple), UM–unmethylated (green), and UM + M (blue) samples.

Our results suggest a significant association of enhancer methylation with *MGMT* promoter methylation, MGMT expression, and clinical and demographic characteristics in glioblastoma. CpGs in intergenic and intragenic enhancers for which we found significant associations are highlighted in Figure 8A and Figure 8B, respectively.

Methylation of different intragenic enhancers strongly positively correlated with each other. Except for a few CpGs, methylation of intragenic enhancers strongly negatively correlated with *MGMT* promoter methylation. Both relationships were observed without sample stratification.

Aran et al. reported that enhancer methylation might predict gene expression levels better than promoter methylation [28]. For enhancer C, identified by Chen et al. [30], located 560 kb upstream of the *MGMT* promoter, we obtained a significant negative correlation between mean methylation and methylation of individual CpGs 05–08, 11–14, and 37–39 with MGMT protein levels for glioblastoma samples expressing MGMT [15]. For none of the other intergenic enhancers, nor for the intragenic ones, did we find such a linear relationship. However, for several CpGs in intragenic enhancers, we identified an inverse L-shaped relationship between methylation status and MGMT expression. At low to intermediate methylation levels, MGMT was not expressed, whereas at high methylation levels, MGMT was expressed. Notably, this finding clearly contrasts with the association between *MGMT* promoter methylation and MGMT expression. Most commonly, low methylation levels of the *MGMT* promoter are sufficient to silence MGMT, and MGMT is only expressed when the promoter is unmethylated. However, a few cases of tumors express MGMT despite promoter methylation and others lack MGMT expression despite promoter unmethylation [14].

Our findings on the inverse L-shaped relationship between intragenic enhancer methylation and gene expression challenge the classical dogma of DNA methylation-dependent gene regulation. The classical hypothesis suggests that increased methylation of CpGs in regulatory elements is associated with gene silencing, while hypomethylation is linked to gene upregulation [34]. In the literature [35], gene body methylation has been associated with higher levels of gene transcription. However, these findings typically refer to gene body CpG islands and not to regions of low CpG density, as is the case with the enhancer regions investigated in this study.

Among enhancers, increased methylation in intragenic enhancers has been particularly associated with suppression of gene expression [36]. It is well known that the methylation status of regulatory elements, including promoters and enhancers, is associated with transcription factor binding. However, the exact mechanism of this interplay remains unclear. Traditionally, methylation of regulatory elements was thought to repress transcription factor binding. However, emerging evidence suggests that transcription factor binding may affect DNA methylation rather than the other way round [37]. Transcription factors can be categorized into three classes: those that preferentially bind unmethylated DNA, those that prefer methylated DNA, and those that are agnostic to the DNA methylation. For the intergenic and intragenic enhancers examined in this study, numerous potential binding sites for transcription factors containing a CG motif were predicted in silico [38]. In vivo, however, the interaction between DNA methylation and transcription factor binding is complex. The sensitivity of transcription factors to methylation of regulatory elements depends on a variety of parameters, including the position of methylated CpGs within their binding motifs [37].

Furthermore, DNA methylation is only one of the factors affecting chromatin structure at regulatory elements and thus transcription factor binding. In addition to DNA methylation, histone modifications have an important impact. Enhancers have been classified as active, primed, poised, or silent [21]. According to the literature, active enhancers are characterized by low DNA methylation, nucleosome depletion, and the presence of active histone modifications such as acetylation of the lysine 27 on histone H3 (H3K27ac) and monomethylation of the lysine 4 on histone H3 (H3K4me1) [19]. However, by investigating the correlation between DNA methylation and chromatin structure, Charlet et al. found co-occurrence of high DNA methylation of CpGs with active histone marks at enhancers [16]. These so-called “bivalent” enhancer regions were proposed to be stabilized by and to require DNA methylation to remain active [16]. The co-existence of repressive DNA methylation and active histone mark H3K27ac at enhancer regions was observed in both normal and cancer cells. Unfortunately, data on histone marks are not available for the sample set analyzed in this study.

*MGMT* promoter methylation serves as a predictive biomarker for the response to TMZ. In this study, we identified an association between mean methylation (CpGs 08–10) and methylation of CpG 08 of enhancer H, and mean methylation (CpGs 01–04, 06–13, and 19–22) of enhancer G with overall survival. Both enhancer F and enhancer G are located in intron 2 of the *MGMT* gene. We have previously reported that methylation of enhancer G (including mean methylation of CpGs 01–03, CpG 03, CpG 07, mean of CpGs 09–13, and CpG 13) is associated with overall survival [15,27]. Among all CpGs targeted, CpG 08 in enhancer F appears to be the strongest potential marker for overall survival prediction. In all cases, patients with lower methylation showed significantly longer overall survival compared to those with higher methylation, based on cut-off values of 50% (this study) or 55% [27]. Notably, neither in this study nor in our previous work, did we find an association between methylation of intergenic enhancers and overall survival.

We did not find any association between methylation of intergenic enhancers and progression-free survival. However, higher methylation levels at several CpGs in intragenic enhancers (CpG 08 in enhancer F, CpGs 06 and 07 in enhancer H), were associated with shorter progression-free survival compared to lower methylation levels. In our previous study, we observed a negative correlation between methylation of CpG 01 in enhancer G and progression-free survival [27]. Notably, all associations with progression-free survival were observed exclusively in samples with unmethylated *MGMT* promoter.

Glioblastoma is a highly aggressive brain tumor with poor prognosis. Yet some patients survive for more than three years post-surgery. Growing evidence suggests that *MGMT* promoter methylation is linked to long-term survival [39,40,41]. However, *MGMT* promoter methylation alone is insufficient for predicting long-term survival. Other factors, such as tumor protein 53 mutation and patient age also seem to play a role [41]. Our patient cohort included samples from three long-term survivors. Tumors of all three patients had a methylated *MGMT* promoter [27], consistent with existing literature. In addition, long-term survival was associated with methylation of intergenic enhancer C, as well as intragenic enhancer G [27]. In this study, we observed significant differences in methylation levels at CpGs in intergenic enhancers A and E, as well as intragenic enhancers F and H, between long-term and short-term survivors. These findings suggest that tumors from long-term survivors may possess a distinct DNA methylation signature that encompasses promoter, intergenic, and intragenic enhancers of *MGMT*. However, given the limited number of long-term survivors in our patient cohort, further studies are necessary to determine whether *MGMT* enhancer methylation can serve as a reliable biomarker for predicting long-term survival. Consistent with our earlier findings, we did not find any meaningful correlation between enhancer methylation and KPS, regardless of whether samples were stratified by the *MGMT* promoter methylation status. This held true for both intergenic and intragenic enhancers.

*TERT* SNPs, such as rs2853669, have been linked to an increased risk of cancer [42]. In this study, we observed significantly higher methylation levels at CpGs located in intergenic enhancers B, D, and E in samples with the mutated genotype (CT) compared to those with the wildtype genotype (TT), without sample stratification. In addition, we found significant associations upon stratification. In *MGMT* promoter methylated glioblastoma samples, significant differences were found for CpGs in intergenic enhancers B and E, as well as intragenic enhancer I. In promoter unmethylated samples with *TERT* SNP mutation, significantly higher methylation levels were observed for intergenic enhancers B, D, and E compared to samples with the *TERT* SNP wildtype. These results are in line with our previous study, where we also reported associations between SNP rs2853669 and intergenic and intragenic enhancer methylation [27]. In addition to *TERT* SNPs, mutations in the promoter of *TERT* have been detected in over 50 cancer types [43]. *TERT* encodes a rate-limiting catalytic subunit of the enzyme telomerase, which maintains genomic integrity through telomere elongation [44]. These mutations result in transcriptional activation of *TERT*, playing a crucial role in tumorigenesis [43]. Glioblastoma frequently exhibit *TERT* mutation C250T or C228T. In our study, we identified significant differences in the methylation of CpG 14 in intergenic enhancer E based on *TERT* promoter genotypes. Recently, we also found associations between methylation of intergenic enhancer C and *TERT* mutations [27].

The intergenic enhancers investigated are located upstream of *MGMT* and downstream of *Ki-67*. *Ki-67* encodes a nuclear protein expressed in all active phases of the cell cycle, but not during the resting phase G0 [45]. Consequently, Ki-67 is widely used as a proliferation marker in various cancer types [46]. Its relevance in glioblastoma is, however, debated [47]. In our study, we found that methylation of three intergenic enhancers was associated with Ki-67 expression. Specifically, several CpGs in intergenic enhancer A, B, and E showed significantly higher methylation levels in samples with high (>50%) Ki-67 index compared to those with low (≤50%) Ki-67 index. In contrast, methylation of intragenic enhancers was not associated with Ki-67 expression. These findings are in line with our previous study, where we exclusively identified associations between Ki-67 expression and methylation in intergenic enhancers, but not with methylation in intragenic enhancers [27].

Without sample stratification, *MGMT* promoter methylation was not associated with the age of the patients at the time of surgery in our patient cohort [27]. However, in the subgroup of tumors with methylated *MGMT* promoter, patients aged ≥ 60 years exhibited significantly higher levels of *MGMT* promoter methylation compared to younger patients (<60 years). In contrast, we found associations between methylation of both intergenic and intragenic enhancers and age, regardless of sample stratification [27]. In this study, significant differences in enhancer methylation between patients <60 years and those ≥60 years were observed for a limited number of CpGs in intergenic enhancers.

At a single CpG in intergenic enhancer B, the methylation status was significantly higher in female patients compared to male patients. This difference was observed in both the analysis of all samples and when focusing specifically on the subgroup with methylated promoter. In our previous study, we also detected sex-specific differences in *MGMT* enhancer methylation for CpGs in enhancer B, enhancer C, as well as CpGs in intragenic enhancer G [27].

Our patient cohort included three individuals for whom immortalized cell cultures could not be established. These samples were previously found to exhibit different methylation levels in enhancers B, C, D, and G, compared to those from primo-cell culture [27]. In this study, we observed significant differences in methylation levels between immortalized and primo-cell cultures across all enhancers, except for intergenic enhancer A. Overall, samples from primo-cell cultures showed significantly higher enhancer methylation compared to those from immortalized cultures. Previous studies have reported that long-term cell culture may lead to alterations in DNA methylation [48,49]. Our findings suggest that DNA methylation alterations are associated with the stability of cell cultures. However, whether these alterations are a cause or a consequence of cell cultivation remains to be elucidated.

Our sample set contained one gliosarcoma sample. Gliosarcoma is a relatively rare primary tumor of the central nervous system involving both malignant glial and sarcomatous elements [50]. Gliosarcoma shares many clinical and genetic characteristics with glioblastoma. However, *MGMT* promoter methylation has been reported to be less frequent compared to glioblastoma [51]. Moreover, treatment with TMZ has been found to be associated with improved overall survival, irrespective of *MGMT* promoter methylation status [51]. In the gliosarcoma sample analyzed in this study, the *MGMT* promoter was highly methylated. Several CpGs in intragenic enhancers exhibited higher methylation levels than most glioblastoma samples with methylated *MGMT* promoter.

In summary, this study, along with previous research [15,27], enhances our understanding of the role and associations of methylation in intergenic and intragenic enhancers of *MGMT* in glioblastoma. However, we would like to stress that our study was performed retrospectively. Further prospective studies on independent and extended cohorts are needed for assessing enhancer methylation as a clinical biomarker. In addition, functional studies, e.g., CRISPR-mediated enhancer deletions, should be performed to confirm causal regulatory effects.

## 4. Materials and Methods

### 4.1. Samples and Cell Culturing

The sample set comprised patient-derived tumor cells (PDCs) from 38 glioma patients, who underwent surgery at the Kepler University Hospital, Department of Neurosurgery between 2001 and 2020 [52]. All adult patients (>18 years) with a diagnosis of glioblastoma were included in the study. Glioblastoma samples were collected consecutively, in the order of the operations and according to the residual quantity of tumor tissue not required for neuropathological processing. We also analyzed the commercially available glioblastoma cell line T98G (ATCC, Manassas, VA, USA). The sample set matched the one analyzed in our previous study [15], with the exception of one glioblastoma sample (GB20), which could not be included due to insufficient sample material. Of the remaining samples, all except one were diagnosed as glioblastoma. One sample was subtyped as gliosarcoma (GS), a rare histopathologic variant of glioblastoma. Samples were classified according to the WHO Classification of CNS tumors published in 2016 [53]. The cohort of samples analyzed consisted of immortalized (GB01–GB19 and GB21–GB35) and primo-cell cultures (GB36–GB38) (Table 1). Cells were cultured as described previously [52] and cell pellets were stored at −80 °C until DNA extraction. Approval for the study was obtained from the local Ethics Commission of the Faculty of Medicine at the Johannes Kepler University of Linz (application E-39-15), and all patients provided written consent.

### 4.2. Determination of MGMT Protein Expression, Ki-67 Index, Genetic Variants, and Clinical Parameters

MGMT protein expression was investigated by Western blot as described by Spiegl-Kreinecker et al. [14]. Ki-67 index, serving as marker of proliferation, was determined during routine diagnostics by immunohistochemistry. Mutations of the *TERT* promoter (C228T, C229A, and C250T) and *TERT* SNP rs2853669 T>C>G genotypes were determined previously by sequencing on a 3130 Genetic Analyzer (Applied Biosystems, Waltham, MA, USA) using BigDye Terminator v1.1 Cycle Sequencing Kit (Applied Biosystems, Waltham, MA, USA) following standard procedures [54]. A list of all clinical and demographic data of the patient cohort is given in Table 1.

### 4.3. DNA Extraction and Bisulfite Conversion

DNA extraction and bisulfite conversion were carried out as previously described [15]. Briefly, genomic DNA was extracted using the QIAamp DNA Blood Mini Kit (Qiagen, Hilden, Germany), according to the manufacturer’s instruction for cultured cells. Bisulfite conversion was performed using the EpiTect Fast Bisulfite Conversion Kit (Qiagen, Hilden, Germany), following the manufacturer’s protocol. DNA quantification was carried out using the Qubit 4 instrument with the Qubit dsDNA BR Assay Kit (for genomic DNA) or the Qubit ssDNA Assay Kit (for bisulfite converted DNA), both from Thermo Scientific, Waltham, MA, USA. DNA was stored at −20 °C.

### 4.4. Primer Design and PCR Conditions for DNA Methylation Analysis

Coordinates for *MGMT* enhancers were obtained from the VISTA Enhancer Browser [29] and nucleotide sequences were retrieved from the National Center for Biotechnology Information (NCBI) database [55] (Supplementary Table S1). Enhancers were named using capital letters based on their genomic position. Enhancers A (hs542 [29]), B (hs737 [29]), C (the enhancer identified by Chen et al. [30], D (hs699 [29]), and E (hs562 [29]) are intergenic enhancers, located between the *Ki-67* and the *MGMT* genes. Enhancers F (hs656 [29]), G (hs696 [29], H (hs331 [29]), and I (hs589 [29]) are intragenic enhancers, with enhancers F, G, and H located in intron 2 and enhancer I being in intron 3 of the *MGMT* gene.

**Figure 9:**
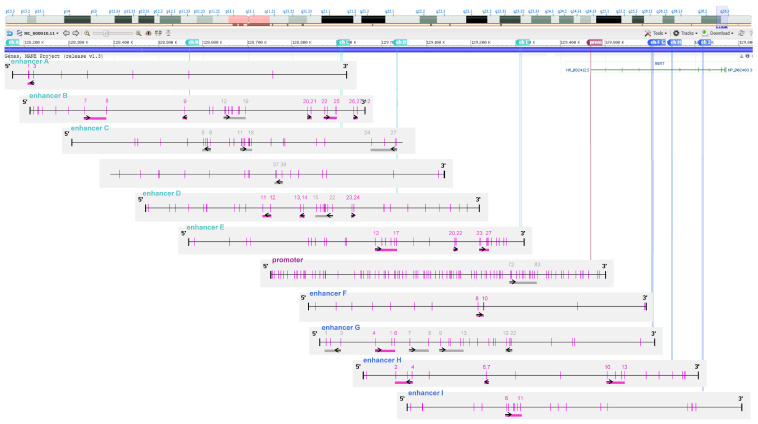
Schematic representation of targeted CpG sites in *MGMT* enhancers and promoter. The figure shows targeted CpG sites in both intergenic (turquoise) and intragenic (blue) *MGMT* enhancers, as well as the *MGMT* promoter, for the current and our previously published study [15]. Pink vertical lines represent CpG sites. Pink and grey horizontal bars indicate CpG sites targeted by individual PSQ assays in the current and previous study, respectively. Black arrows denote the sequencing direction relative to the upper strand. CpG sites are numbered according to their positions in the respective regulatory element. CpG representations were generated with Methyl Primer Express Software v1.0 (Thermo Scientific, Waltham, MA, USA) and manually modified. Chromosome 10 representation, including the *MGMT* gene location, was sourced from the NCBI Genome Data Viewer [56]. Details of the enhancers are as follows. Intergenic enhancers: enhancer A (hs542): 1032 bp, 8 CpGs; enhancer B (hs737): 1138 bp, 27 CpGs; enhancer C (Chen et al. [30]: 3313 bp, 46 CpGs, CpGs 1–25: “Del 1”, CpGs 26–46: “Del2”; enhancer D (hs699): 1719 bp, 33 CpGs; enhancer E (hs562): 2221 bp, 32 CpGs. Intragenic enhancers: enhancer F (hs656): 1332 bp, 12 CpGs; enhancer G (hs696): 1243 bp, 26 CpGs; enhancer H (hs331): 1899 bp, 20 CpGs; enhancer I (hs589): 1366 bp, 21 CpGs.

We designed 18 assays targeting CpG sites in *MGMT* enhancers, illustrated in Figure 9, using PyroMark Assay Design Software 2.0.1.15 (Qiagen, Hilden, Germany). Primer sequences for these assays are given in Supplementary Table S1.

Optimization of annealing and elongation temperatures, as well as primer concentrations, was performed for each assay using bisulfite converted human DNA standards (Zymo Research, Irvine, CA, USA). Each reaction setup consisted of 1× PyroMark Mastermix, 1× CoralLoad concentrate, EvaGreen HRM dye, forward and reverse primers, and either 2.5 or 5 ng of bisulfite converted DNA sample, resulting in a total volume of 20 µL. In each PCR run, two no-template controls, where DNA was replaced by nuclease-free water, were included. Following amplification, high-resolution melting (HRM) analysis was carried out to monitor side product formation. For assay optimization, the QuantStudio 5 thermal cycler (Thermo Scientific, Waltham, MA, USA) was used. For identity, quality, and yield assessment, amplicons were subjected to gel electrophoresis on a 3% agarose gel in 0.5× TBE (Tris-borate-EDTA) buffer. Bands were visualized with a UVT-20 M transilluminator (Herolab, Wiesloch, Germany). PCR for sample analysis was performed on the RotorGene Q instrument with a 72-well rotor (Qiagen, Hilden, Germany).

### 4.5. PSQ of PCR Products

PCR products were sequenced using the PyroMarkQ48 Autoprep instrument, along with PyroMarkQ48 Autoprep Accessories, PyroMarkQ48 Advanced CpG Reagents, and PyroMarkQ48 Autoprep Software 2.4.2, following the manufacturer’s instructions (all from Qiagen, Hilden, Germany). Volumes of 8–10 µL of PCR products were used for sequencing. Each sample was analyzed in duplicate, by performing two independent PCR and PSQ runs.

### 4.6. Data Analysis and Statistics

PCR amplification and HRM curves were acquired using RotorGene Q Series Software 2.3.1 (Qiagen, Hilden, Germany). Pyrograms were evaluated using PyroMarkQ48 Autoprep Software 2.4.2 (Qiagen, Hilden, Germany), and methylation data were exported. Data analysis and graphical presentation of results were carried out using R Statistical Software (version 4.3.1; R Core Team 2023). R packages used are listed in Supplementary File R-packages.

To assess significant differences between two groups, a Student’s *t*-test was carried out. In the case of more than two groups, one-way ANOVA (analysis of variance) followed by post hoc *t*-test corrected for multiple testing was performed. Only groups with more than one member were included in the analysis. To assess correlations between two variables, Pearson’s correlation coefficients as well as scatterplots were used, without correcting for multiple testing. For analysis of survival data, Kaplan–Meier plots were generated using log-rank test. For statistical significance, *p*-values ≤ 0.05 were regarded as statistically significant. Statistical analysis was confined to PDCs from glioblastoma patients (GB01–19 and GB21–38). Results for T98G as well as the gliosarcoma sample were only discussed in a descriptive manner. In the case of clinical and demographic parameters, statistical analysis was carried out excluding the samples GB36–38, for which no immortalized cell cultures could be obtained. To obtain a more comprehensive view, methylation levels obtained in this study and in our previous study [15] were included.

## 5. Conclusions

Altogether, in this study and our previous work, we targeted a total of 105 CpGs in five intergenic and four intragenic *MGMT* enhancers, applying in-house developed PSQ assays.

Our results suggest a relationship between methylation levels and the relative position of the CpGs within intergenic enhancers, with CpGs closer to the 5′ end being significantly more methylated compared to those closer to the 3′ end. We also observed an association between enhancer methylation and *MGMT* promoter methylation. Samples with unmethylated *MGMT* promoter showed significantly higher methylation levels in both intergenic and intragenic enhancers than *MGMT* promoter methylated samples.

In addition, our findings indicate an inverse L-shaped relationship between methylation of intragenic enhancers and MGMT expression. Data on transcription factor binding and the presence of histone marks at the enhancer regions in this sample set would provide further insights into the activity of these enhancers.

Our results on clinical and demographic parameters corroborate with previous findings [27], indicating that *MGMT* enhancer methylation is associated with genetic variants, clinical parameters, and demographic characteristics in glioblastoma. Specifically, methylation levels of intergenic enhancers were primarily linked to the *TERT* SNP rs2853669 genotype, Ki-67 expression, age, and sex, while those of intragenic enhancers were associated with *MGMT* promoter methylation, MGMT expression, overall survival, and progression-free survival. Among all CpGs analyzed, CpG 08 in enhancer F appears to be the most promising biomarker for predicting overall survival.

Patients who survived more than three years post-surgery, termed long-term survivors, exhibited significantly different methylation levels in three intergenic and three intragenic enhancers compared to short-term survivors.

Growing evidence suggests that long-term cell culture may lead to alterations in DNA methylation. In our study, samples from primo-cell cultures showed significantly higher methylation levels across all enhancers investigated, except for intergenic enhancer A, compared to immortalized ones, indicating that cell culture stability is associated with changes in DNA methylation.

We observed some associations without stratifying samples by their *MGMT* promoter methylation status, while others were found in subgroups (either methylated or unmethylated *MGMT* promoter samples), underscoring the importance of *MGMT* promoter methylation in glioblastoma.

Since our findings are based on a relatively small and heterogenous sample set, further studies are necessary to validate our results.

## Figures and Tables

**Table 1:** Clinical and demographic data of GB01–GB19, GB21–38, and GS01.

Patient	Age [y]	Sex	KPS [%]	Ki-67 [%]	OS [m]	PFS [m]	MGMT Exp	*TERT* Prom	*TERT* rs2853669
GB01	64	f	60	n.s.	7.43	4.00	0.00	C250T	TT
GB02	85	m	40	≤50 (30)	3.00	n.s.	0.00	wt	TT
GB03	53	f	100	n.s.	52.50	4.50	0.00	C228T	CT
GB04	67	f	70	>50 (90)	46.63	0.53	0.00	C228T	TT
GB05	57	f	100	≤50 (30)	10.50	6.00	0.00	C228T	TT
GB06	46	m	90	n.s.	30.50	4.00	0.00	C228T	TT
GB07	50	f	90	n.s.	27.40	n.s.	0.00	C250T	n.s.
GB08	74	f	70	≤50 (30)	7.79	n.s.	0.00	C228T	CT
GB09	48	m	60	n.s.	1.55	n.s.	0.00	wt	TT
GB10	64	m	80	>50	11.70	n.s.	0.20	C228T	CT
GB11	73	f	60	>50	10.60	n.s.	0.16	C228T	TT
GB12	44	m	80	>50	13.00	9.00	1.10	C250T	CT
GB13	65	m	90	≤50 (15)	9.27	n.s.	1.20	C228T	TT
GB14	69	m	90	≤50 (40)	8.00	n.s.	0.04	C250T	TT
GB15	73	f	100	n.s.	7.00	2.63	1.10	C250T	CT
GB16	83	m	90	≤50	9.57	9.00	1.00	C228T	CT
GB17	74	m	90	n.s.	5.19	n.s.	1.32	C250T	CT
GB18	44	m	100	n.s.	23.15	n.s.	0.25	wt	TT
GB19	48	m	100	>50 (60)	18.67	4.54	0.09	C228T	CT
GB21	60	m	90	≤50 (40)	13.00	3.00	0.40	C228T	TT
GB22	53	m	70	>50 (60)	0.89	n.s.	0.54	C250T	TT
GB23	47	f	70	>50 (70)	37.25	8.00	0.00	C250T	CT
GB24	64	m	90	≤50 (40)	7.73	n.s.	0.49	C228T	CT
GB25	67	f	70	>50	16.80	10.00	0.00	C228T	TT
GB26	75	f	70	≤50 (40)	8.22	n.s.	0.00	C228T	CT
GB27	52	f	90	>50 (70)	n.s.	n.s.	0.00	C250T	TT
GB28	63	m	n.s.	>50	21.80	8.00	0.00	C250T	CT
GB29	79	f	40	>50	1.31	n.s.	0.00	C228T	CT
GB30	58	m	90	>50	16.00	4.31	0.00	C228T	TT
GB31	58	f	n.s.	>50	13.30	4.00	0.00	C228T	TT
GB32	71	m	n.s.	≤50 (20)	1.25	n.s.	0.00	C228T	TT
GB33	57	m	n.s.	n.s.	12.70	6.00	0.20	C250T	CT
GB34	53	m	n.s.	n.s.	32.30	22.00	0.00	C228T	TT
GB35	64	m	40	n.s.	10.60	n.s.	0.00	C228T	TT
GB36	62	m	50	≤50 (25)	1.48	n.s.	0.90	wt	TT
GB37	63	m	80	>50 (60)	19.50	9.01	0.11	wt	TT
GB38	76	m	30	≤50 (20)	4.44	n.s.	0.29	wt	CT
GS01	43	f	80	≤50 (30)	9	n.s.	0.00	C250T	TT

GB01–19 and GB21–38: patients with glioblastoma (GB36–GB38: no immortalized cell cultures obtained); GS01: patient with gliosarcoma; numbering of samples was adapted from our previous study [15]. exp: expression, f: female, KPS: Karnofsky Performance Score, m (sex): male, m: months, n.s.: not specified, OS: overall survival, PFS: progression-free survival, prom: promoter, wt: wildtype, y: years.

## Data Availability

The original contributions presented in this study are included in the article/Supplementary Materials. Further inquiries can be directed to the corresponding author(s).

## Supplementary Materials

The supporting information can be downloaded at https://www.mdpi.com/article/10.3390/ijms26073390/s1. References [57,58,59,60,61,62,63,64,65] are cited in the supplementary materials.

## Abbreviations

The following abbreviations are used in this manuscript:

CpG	CpG dinucleotide
EGFR	epidermal growth factor receptor
GB	Glioblastoma
GS	Gliosarcoma
HRM	High-resolution melting
IDH	Isocitrate dehydrogenase
KPS	Karnofsky Performance Score
MGMT	O6-methylguanine-DNA methyltransferase
NCBI	National Center for Biotechnology Information
OS	Overall survival
PCR	Polymerase chain reaction
PDCs	Patient-derived cells
PSQ	Pyrosequencing
TERT	Telomerase reverse transcriptase
TMZ	Temozolomide
WHO	World Health Organization
