# Supplementary material for: Association of Intergenic and Intragenic MGMT Enhancer Methylation with MGMT Promoter Methylation, MGMT Protein Expression and Clinical and Demographic Parameters in Glioblastoma
Source: Int J Mol Sci. 2025 Apr 4;26(7):3390. doi: 10.3390/ijms26073390 (PMC11990025; doi:10.3390/ijms26073390)
Supplement: Supplementary file 1 [file ijms-26-03390-s001.zip › Supplementary Figures.pdf]

*Supplementary Figures*

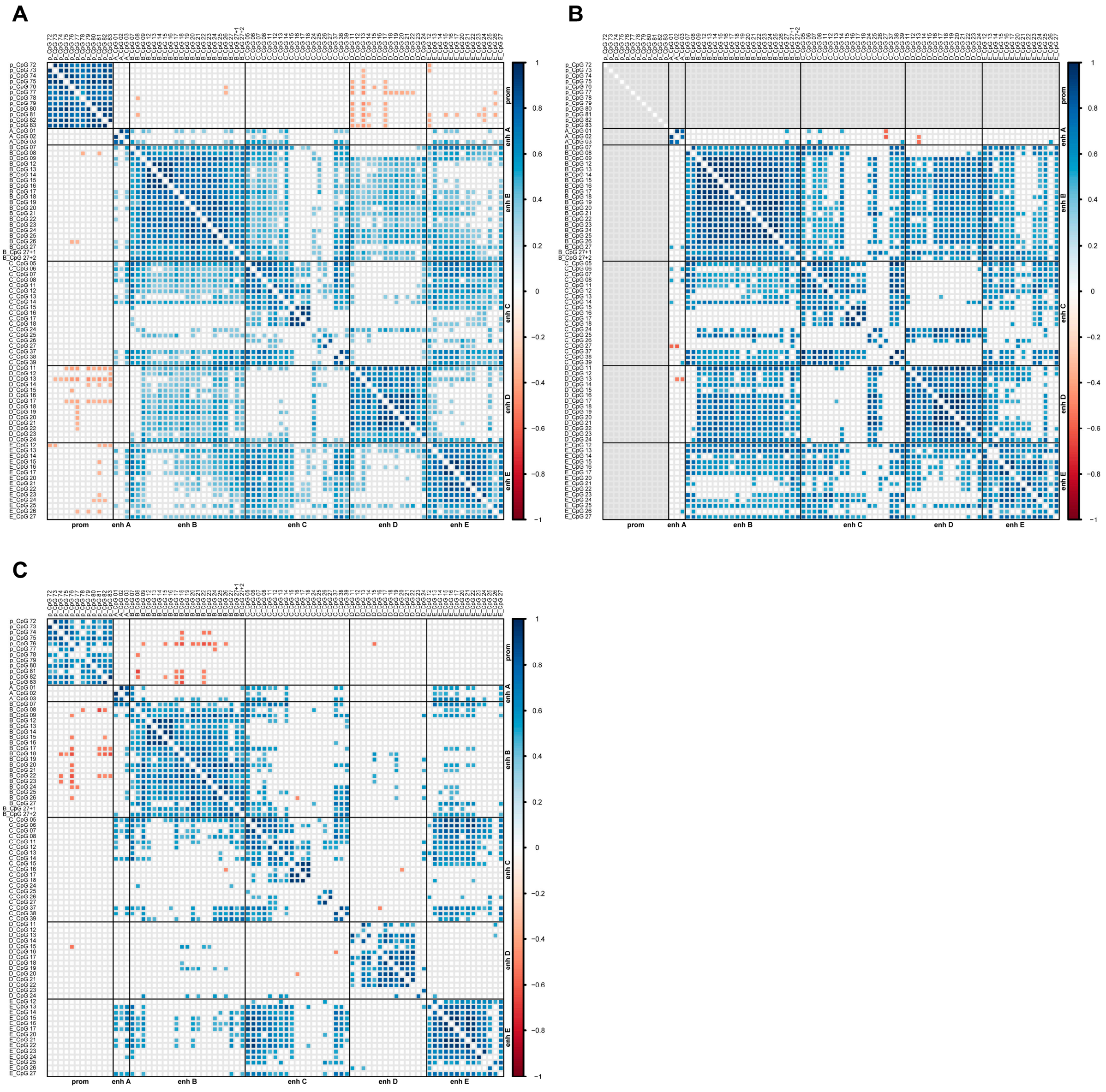


**Figure S1.** Correlation plots displaying significant Pearson's correlation coefficients between methylation levels of individual CpGs in intergenic *MGMT* enhancers and the *MGMT* promoter (CpGs 72–83). CpG methylation levels from this study and from our previous study were included [1]. The color scale ranges from dark red (-1.0) to dark blue (1.0). Grey tiles indicate not analyzable data. Plots are shown for **(A)** all, **(B)** *MGMT* promoter unmethylated, and **(C)** *MGMT* promoter methylated IDH-wildtype glioblastoma (GB01–19 and GB21–38). Correlation analysis was carried out using pairwise complete comparisons.

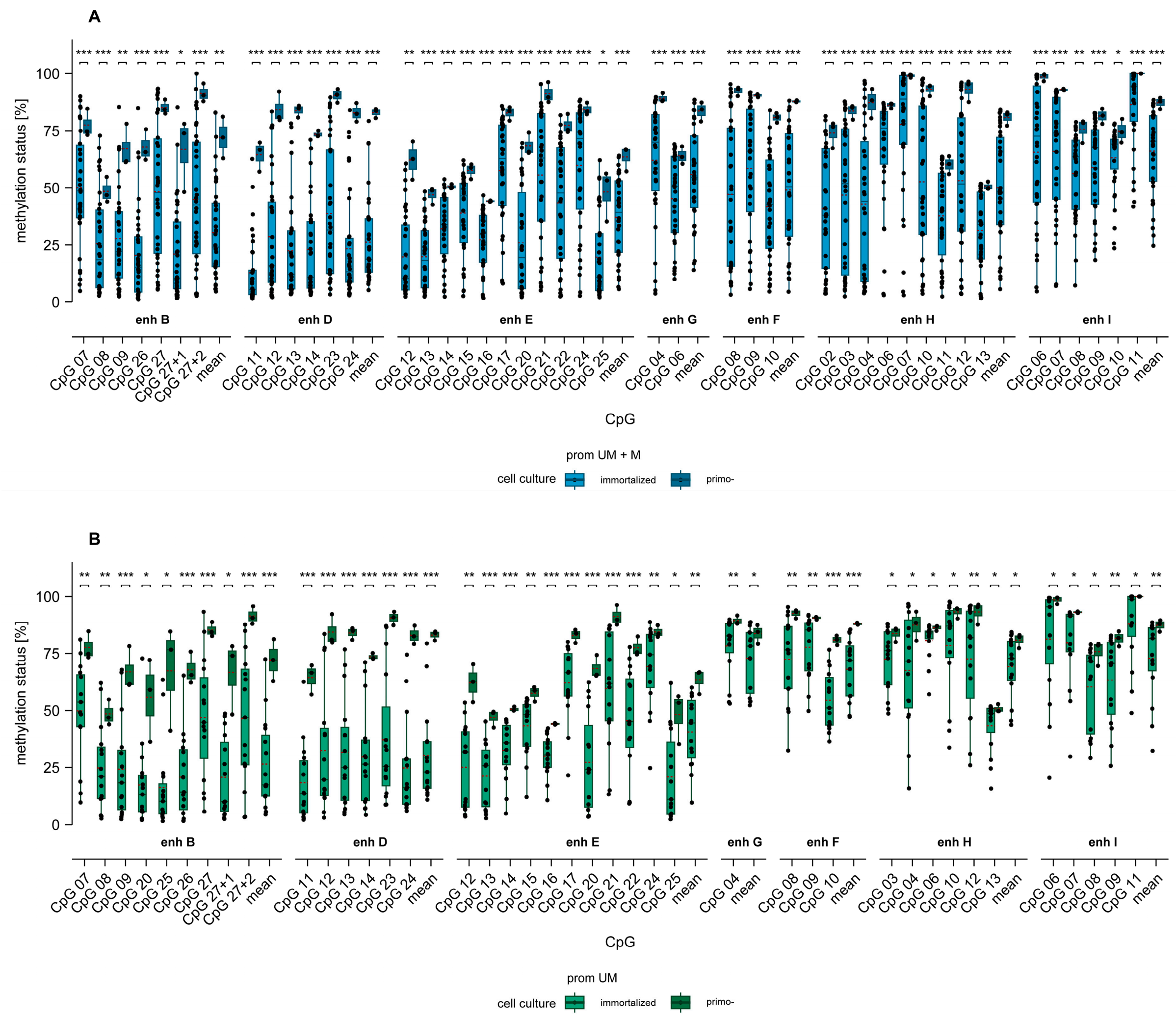


**Figure S2.** Significantly different *MGMT* enhancer methylation levels between immortalized and primo- cell cultures in **(A)** all and **(B)** promoter unmethylated glioblastoma samples. The red dashed line represents the mean methylation level. Significance marks: $*p \leq 0.05$, $**p \leq 0.01$, $***p \leq 0.001$.

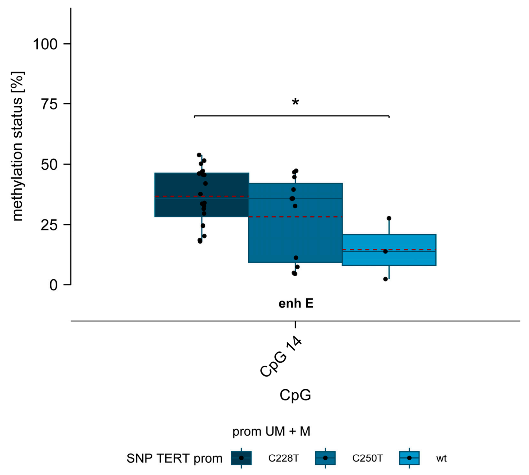

**Figure S3.** Significantly different *MGMT* enhancer methylation levels between samples with *TERT* promoter wildtype and 228T and C250T mutations. The red dashed line represents the mean methylation level. Significance mark: *$p \leq 0.05$.


1. Zappe, K.; Pühringer, K.; Pflug, S.; Berger, D.; Böhm, A.; Spiegl-Kreinecker, S.; Cichna-Markl, M. Association between MGMT Enhancer Methylation and MGMT Promoter Methylation, MGMT Protein Expression, and Overall Survival in Glioblastoma. *Cells* **2023**, *12*, doi:10.3390/cells12121639.
