# Supplementary material for: Association of Intergenic and Intragenic MGMT Enhancer Methylation with MGMT Promoter Methylation, MGMT Protein Expression and Clinical and Demographic Parameters in Glioblastoma
Source: Int J Mol Sci. 2025 Apr 4;26(7):3390. doi: 10.3390/ijms26073390 (PMC11990025; doi:10.3390/ijms26073390)
Supplement: Supplementary file 1 [file ijms-26-03390-s001.zip › Supplementary File S1_R_packages.pdf]

R-packages

Wickham et al. (2019). Welcome to the tidyverse. Journal of Open Source Software, 4(43), 1686. https://doi.org/10.21105/joss.01686.

Taiyun Wei and Viliam Simko (2021). R package 'corrplot': Visualization of a Correlation Matrix (Version 0.92). https://CRAN.R-project.org/package=corrplot

Hadley Wickham (2016). ggplot2: Elegant Graphics for Data Analysis. Springer-Verlag New York. https://ggplot2.tidyverse.org

Alboukadel Kassambra (2023). ggpubr: 'ggplot2' Based Publication Ready Plots. R-package version 0.6.0. https://CRAN.R-project.org/package=ggpubr

William Revelle (2024). psych: Procedures for Psychological, Psychometric, and Personality Research. Northwestern University, Evanston, Illinois. R package version 2.4.6. https://CRAN.R-project.org/package=ggsignif

Baptiste Auguie. (2017). gridExtra: Miscellaneous functions for “grid” graphics (Version 2.3). https://CRAN.R-project.org/package=gridExtra

David Robinson, Alex Hayes and Simon Couch (2022). broom: Convert Statistical Objects into Tidy Tibbles. R package version 1.0.6. https://CRAN.R-project.org/package=broom

Winston Chang W (2023). extrafont: Tools for Using Fonts. R package version 0.19. https://CRAN.R-project.org/package=extrafont

Terry Therneau (2024). A Package for Survival Analysis in R. R package version 3.5-8. https://CRAN.R-project.org/package=survival
