# Supplementary material for: Association of Intergenic and Intragenic MGMT Enhancer Methylation with MGMT Promoter Methylation, MGMT Protein Expression and Clinical and Demographic Parameters in Glioblastoma
Source: Int J Mol Sci. 2025 Apr 4;26(7):3390. doi: 10.3390/ijms26073390 (PMC11990025; doi:10.3390/ijms26073390)
Supplement: Supplementary file 1 [file ijms-26-03390-s001.zip › Supplementary Table.pdf]

Supplementary Tables

Table S1. Clinical and demographic data of GB01–GB19, GB21–38 and GS01.

Enhancer	Coordinates	CpGs analyzed	Primer sequence (5′→3′)	Amplicon length [bp]
**A**	NC_000010.11:128165799 –128166830	01-03	F: TGTTGGTTATTGTGAGTGATAGT	164
(hs542)			R: [Btn] AAACTCCATCCACTAAAACCTA	
8 CpGs			S: ATTTTTTGTTATGTAAAGT	
**B**	NC_000010.11:128568604 –128569741	07, 08	F: AGGTGGYAGAGAGAATG	147
(hs737)			R: [Btn] ACAAARTCATTATCCCATCT	
27 CpGs			S: AGGTGGYAGAGAGAATG	
		09	F: [Btn] GTAGGTAGGGTTTGTGTAGTT	121
			R: TTAACTAATCCCAAATCTACATTCT	
			S: TCTACATTCTCTTCT	
		20, 21	F: AGGGATTTGGAGGGTAGGTGAA	97
			R: [Btn] TCAACTATCTACTTAACACAAACCAC	
			S: AGTTGTGTTTGTTTAGG	
		22-25	F: GTTGAGTGTGGTTTGTGTTAAGT	136
			R: [Btn] ACAACCTACCTCCTCTATACC	
			S: GTTAAGTAGATAGTTGA	
		26,27 +2	F: GGGTTTTGTGGAGGAATAATAAA	190
			R: [Btn] CTTTATACCTACAACATACTCCTA	
			S: TTTAGGGTATAGAGG	
**D**	NC_000010.11:129033193 –129034911	11, 12	F: AATTAAATTGTTAAGTAGGTATTAGAG	141
(hs699)			R: [Btn] AAATATCAAACTCTCAAATCATCCT	
33 CpGs			S: TAGGTATTAGAGGTTG	
		13, 14	F: TTGGTTTGTGTGTTATTTATAGTAT	169
			R: [Btn] TCCTCAATATTCAAACTATCATAATA	
			S: GTTGAATTTAGTTTTTGTAA	
		23, 24	F: TGTAAATAAGTAAAAAAAAGGGY	240
			R: [Btn] AACCAATCTCTCCCTCC	
			S: TTTGTTGAYAGAAGTGG	
**E**	NC_000010.11:129308258 –129310478	12-17	F: TGTGGTAGTGGTATTTTTTAGTTAGTAGA	268
(hs562)			R: [Btn] CCATTTTAATATACAACACTTTCCCTTTTT	
32 CpGs			S: AATTATTTTTTATGATTGTTA	
		20-22	F: TTTGTATATTGAAGAGGGAAGAAGAA	134
			R: [Btn] AAAAAAAATAATTACTCTAACAACCCTTT	
			S: ATTATTTTTTATATTTTAG	
		23-27	F: ATTGTAAGGGAAAGGGTTGTTAGAGTAA	221

Enhancer	Coordinates	CpGs analyzed	Primer sequence (5′→3′)	Amplicon length [bp]
			R: [Btn] AACTCCCCTACAAACTTTTCACCCTAC	
			S: AGGTTTTGTTTATTGTAATTTTATG	
**F** (hs656) 12 CpGs	NC_000010.11:129602684 –129604015	08-10	F: TTAATGATTTTTAGTTGTTTGTTGT	198
			R: [Btn] TACTATTATTATACATATCCAAATAAA	
			S: AGATGATTAGTAAGTGAGA	
**G** (hs696) 26 CpGs	NC_000010.11:129605804 –129607046	04, 06	F1: TTGTTGTGGGATTTGTGAG	147
			R1: [Btn] CACATACCATCATTACTTAT	
			R2: [Btn] CACATGCCATCATTACTTAT	
			S: TTGTGGGATTTGTGAG	
**H** (hs331) 20 CpGs	NC_000010.11:129647523 –129649421	02-04	F: GGGTTGTTAGAAAGTTGATGAAA	180
			R: [Btn] AAAAAAAACAATTCATCTCCCTTTAT	
			S: AGAAAGTTGATGAAATG	
		06, 07	F: ATTGAATTATGGGTTTATTTAAAATGGT	249
			R: [Btn] ACACTTTCCACCTTTTACTATCA	
			S: GAGGTTAAAATTTTTATAGATTGGA	
		10-13	F: GATTTTTTAAATTAGTGAAGAGTGGATATT	182
			R: [Btn] AAATCCCACAACCACAAAAAAAAACAA	
			S: TTAAATTAGTGAAGAGTGGATATT	
**I** (hs589) 21 CpGs	NC_000010.11:129716557 –129717922	06-11	F: TGGTTTAAGTTATTTAGGTTGAATGTTAAT	209
			R: [Btn] CTACTCATTAAAAATACCATCACTCAAAT	
			S: ATTGAGTATATTTTATTAAGAA	

[Btn]: biotin; length: PCR product length; bp: base pairs; F: forward primer; R: reverse primer; S: sequencing primer; Y: C/T
